# Immune trypanolysis test as a promising bioassay to monitor the elimination of *gambiense* human African trypanosomiasis

**DOI:** 10.1051/parasite/2019066

**Published:** 2019-11-22

**Authors:** Emilie Dama, Oumou Camara, Dramane Kaba, Mathurin Koffi, Mamadou Camara, Charlie Compaoré, Hamidou Ilboudo, Fabrice Courtin, Jacques Kaboré, Emmanuel Kouassi N’Gouan, Philippe Büscher, Veerle Lejon, Bruno Bucheton, Vincent Jamonneau

**Affiliations:** 1 Centre International de Recherche-Développement sur l’Elevage en zones Subhumides (CIRDES), Unité de recherches sur les maladies à vecteurs et biodiversité 01 BP 454 Bobo-Dioulasso 01 Burkina Faso; 2 Université Nazi Boni, Bobo-Dioulasso 01 BP 1091 Bobo-Dioulasso Burkina Faso; 3 Programme National de Lutte contre la Trypanosomose Humaine Africaine BP 851 Conakry Guinée; 4 Institut Pierre Richet, Unité de Recherche « Trypanosomoses » 01 BP 1500 Bouaké Côte d’Ivoire; 5 Université Jean Lorougnon Guédé, Laboratoire de biodiversité et Gestion durable des écosystèmes tropicaux, Unité de Recherche en Génétique et Epidémiologie moléculaire BP 150 Daloa Côte d’Ivoire; 6 Institut de Recherche en Sciences de la Santé (IRSS), Unité de Recherche Clinique de Nanoro (URCN) 11 BP 218 Ouagadougou CMS 11 Burkina Faso; 7 Institut de Recherche pour le Développement (IRD), UMR INTERTRYP IRD-CIRAD, Université de Montpellier, TA A-17/G, Campus International de Baillarguet F-34398 Montpellier France; 8 Projet de Recherche Clinique sur les Trypanosomoses BP 1425 Daloa Côte d’Ivoire; 9 Institute of Tropical Medicine, Department of Biomedical Sciences Nationalestraat 155 2000 Antwerp Belgium

**Keywords:** Human African Trypanosomiasis, *Trypanosoma brucei gambiense*, Elimination, Diagnosis, Transmission, Immune trypanolysis

## Abstract

The World Health Organization (WHO) has set the goal of *gambiense*-Human African trypanosomiasis (HAT) elimination as a public health problem for 2020 and interruption of transmission in humans for 2030. In this context, it is crucial to monitor progress towards these targets using accurate tools to assess the level of transmission in a given area. The aim of this study was to investigate the relevance of the immune trypanolysis test (TL) as a population-based bioassay to evaluate *Trypanosoma brucei gambiense* transmission in various epidemiological contexts. Significant correlations were observed between HAT endemicity levels and the percentage of TL-positive individuals in the population. TL therefore appears to be a suitable population-based biomarker of the intensity of transmission. In addition to being used as a tool to assess the HAT status at an individual level, assessing the proportion of TL positive individuals in the population appears as a promising and easy alternative to monitor the elimination of *gambiense* HAT in a given area.

## Abstract (fr)

L’Organisation mondiale de la santé a fixé comme objectif l’élimination de la trypanosomose humaine africaine (THA) à *gambiense* en tant que problème de santé publique à l’horizon 2020 et l’interruption de la transmission humaine pour 2030. Dans ce contexte, il est crucial de suivre les progrès accomplis vers ces objectifs à l’aide d’outils précis pour évaluer le niveau de transmission dans une zone donnée. Le but de ce travail était d’étudier la pertinence du test immunitaire de trypanolyse (TL) en tant que marqueur biologique populationnel pour évaluer la transmission de *Trypanosoma brucei gambiense* dans divers contextes épidémiologiques. Des corrélations significatives ont été observées entre les niveaux d’endémicité de la THA et le pourcentage d’individus positifs à la TL dans la population. La TL apparaît donc comme un biomarqueur populationnel de l’intensité de la transmission. En plus d’être utilisé comme un outil pour évaluer le statut de la THA au niveau individuel, l’évaluation de la proportion d’individus positifs à la TL dans la population apparaît comme une alternative simple et prometteuse pour surveiller l’élimination de la THA à *gambiense* dans une zone donnée.

## Introduction

Human African trypanosomiasis (HAT) or sleeping sickness is an infectious disease caused by protozoan parasites belonging to the species *Trypanosoma brucei* and affecting rural populations in sub-Sahara Africa. The chronic form of the disease, due to *Trypanosoma brucei gambiense*, occurs in West and Central Africa and accounts for over 97% of current cases [3]. Thanks to considerable control efforts, less than 10,000 new cases per year have been reported since 2009, and 1447 cases of HAT were reported in 2017. The World Health Organization (WHO) has set the goal of *gambiense*-HAT elimination as a public health problem for 2020 and interruption of transmission in humans for 2030 [18].

Control of *gambiense-*HAT primarily relies on case detection and treatment. For active case detection, mobile teams screen the population at risk for the presence of antibodies with the Card Agglutination Test for Trypanosomiasis (CATT/*T.b. gambiense*, [33]) and examine CATT-seropositive subjects for parasitological confirmation by microscopy. Passive case detection or passive surveillance in peripheral health centres is based on rapid diagnostic tests (RDTs) for antibody detection, [2, 4] followed by parasitological examinations on RDT-positive subjects.

As for most serological tests, the specificity of CATT and RDTs is not 100%, leading to false-positive subjects and low to very low positive predictive values in low endemic areas. On the other hand, parasitological methods are not 100% sensitive, leading to false-negative subjects [8, 26]. A test that is considered 100% specific for antibodies against *T.b. gambiense* is the immune trypanolysis test (TL) using the variant antigen types (VAT) LiTat 1.3 and LiTat 1.5 [25, 39]. It is used to identify among the parasitologically unconfirmed seropositive subjects, those who are or have been in contact with *T.b. gambiense* [4, 6, 14, 19, 21–24, 26, 27, 29, 32, 34]. TL is now also implemented in some diagnostic algorithms for *gambiense*-HAT [25, 40]. Adaptation of TL for testing blood-impregnated filter paper has facilitated specimen storage and shipment to reference laboratories [6].

The indicators to monitor progress towards the 2020 and 2030 WHO targets are based on the number of reported cases, the related areas and populations exposed at various levels of risk, and the coverage of surveillance activities [18]. The aim of this study was to investigate the relevance of TL as a population-based bioassay to assess *T.b. gambiense* transmission in active or extinct HAT foci.

## Materials and methods

### Ethics statement

All samples were collected within the framework of medical surveys and epidemiological surveillance activities supervised by the HAT National Control Programmes. No samples other than those for routine screening and diagnostic procedures were collected. All participants were informed of the objectives of the study in their own language and signed an informed consent form. This study is part of a project aiming to improve HAT diagnosis for which approval was obtained from the WHO (Research Ethics Review Committee) and the Institut de Recherche pour le Développement (Comité Consultatif de Déontologie et d’Éthique) ethics committees. According to national procedures, CATTP and TL-positive subjects negative to parasitological examinations are followed-up until CATTP becomes negative or treated in case parasitology becomes positive during the follow-up.

### Description of the study sites and sampling

For the purpose of this study, we selected all plasma specimens that were collected by our team between 2010 and 2014 during mass screening campaigns led in West Africa with the National Control Programmes, and during which plasma samples were systematically collected from all tested individuals.

In Guinea, the Boffa focus is located in a mangrove area of coastal Guinea. It is currently the most active West African focus with an average prevalence of about 0.5%. The vector is *Glossina palpalis gambiensis* [10]. From this focus, 1166 subjects previously sampled in 2012 and 2013 and for whom CATT and parasitological results were available [10, 13] were included in the present analysis.

In Côte d’Ivoire, the study was carried out in the *gambiense*-HAT endemic foci of Sinfra and Bonon, located in the west-central part of the country, where the forest has been progressively replaced by cash crops (mainly cocoa and coffee) leading to a favorable environment for *G. palpalis palpalis* and HAT transmission [31]. Control efforts conducted from 1992 to the present day have largely contained the epidemic in Sinfra (1992–1997) and in Bonon (1998–2004) [15, 28, 30]. However, few cases are still diagnosed each year [30, 32]. From Bonon and Sinfra, 283 and 341 subjects were sampled in 2014, respectively.

In Côte d’Ivoire, part of the study was also carried out in the village Boblénou near Bouaké in the center of the country. Bouaké is a commercial city with a large population mix coming from all over the country, but also from neighbouring countries (mainly Burkina Faso and Mali) since the beginning of the 20th century. The consequence regarding HAT is that most of the cases previously reported from this area were probably not due to local transmission but were infections contracted in other areas [1]. This was probably also the case with the last four HAT patients reported from Bouaké in the 1990s [15] who all stayed for longer periods in an active HAT focus (Sinfra and Daloa) before their diagnosis (Ministry of Health, archive was consulted for this circumstance). Nonetheless, even though there is no clear evidence from the literature on active local transmission in the Bouaké area, this cannot be excluded since *G. palpalis palpalis* has always been present in this area [7, 20, 35]. From Boblénou, 192 subjects were sampled in 2016.

In Burkina Faso, the study included the Gaoua historical *gambiense*-HAT foci [16], where no HAT cases have been reported over the last 20 years while medical survey activities were conducted between 2005 and 2008 [11]. However, this historical focus is located in an agro-pastoral area in the South-Western part of Burkina Faso where a risk of re-emergence of HAT still exists due to (i) significant population movements between this area and HAT endemic foci in Côte d’Ivoire [30, 31], (ii) favourable conditions for two tsetse fly species that transmit *T.b. gambiense* (*Glossina palpalis gambiensis* and *G. tachinoides*) [36], and (iii) high human/vector contact [12]. From Gaoua, 729 subjects were sampled in 2014.

All subjects were sampled within the framework of exhaustive mass-screening campaigns that were held during the 2010–2014 period, and during which all inhabitants were invited to participate. In the Bouaké area, active screening was performed in a single village (Boblénou), whereas in the Boffa, Sinfra, Bonon and Gaoua areas, active screening was performed in several villages considered at high risk. No children below the age of five were sampled in any of the study sites. For sampling, two capillary tubes were collected from each participant. The first one was used to perform the CATT on whole blood (CATTB) in the field. For CATTB-positive individuals, blood was collected in heparinised tubes and a twofold plasma dilution series in CATT buffer was tested to assess the end titer, i.e., the highest dilution still positive on plasma (CATTP). All CATTP ≥ 1/4 (CATTP+) underwent parasitological examinations by direct examination of a lymph node aspirate in case of presence of cervical lymphadenopathy and/or by the mini-anion exchange centrifugation technique on buffy coat (mAECT-BC) [5]. The second capillary tube was centrifuged (12,500 rpm for 5 min) and 25 μL of plasma were collected and kept at −20 °C for subsequent TL testing.

In total, 2711 persons were included in this analysis. Table 1 summarizes the sampling and presents information about the occurrence of tsetse fly species and the number of HAT cases reported between 2010 and 2014 (including the sampling period) in each study area derived from the WHO atlas for HAT [37]. In line with the WHO expert committee recommendations regarding validation of HAT elimination by endemic countries [18], HAT endemicity was calculated as the mean number of reported cases per 10,000 inhabitants over the 2010–2014 5-year period for each study site. The districts of Gaoua in Burkina Faso and Boblénou in Côte d’Ivoire reported no cases in the last 20 years and we may consider that there is no more active transmission of *T.b. gambiense* to humans in these areas (endemicity is 0 cases/10,000 inhabitants/year). With respectively 5 cases (0.15 cases/10,000 inhabitants/year) and 19 cases (0.47 cases/10,000 inhabitants/year) in 2010–2014, Sinfra and Bonon can be considered as low endemic foci with residual transmission that have reached the 2020 target of reporting less than one HAT case per 10,000 inhabitants. With 2.41 cases/10,000 inhabitants/year (169 cases in 2010–2014), the Boffa focus was still above the elimination threshold and represents a disease focus where transmission is still active.

**Table 1:** Study sites and samples taken.

Country	Area/Focus	Year of sampling	Nb samples	Tsetse species	HAT cases 2010–2014α	Estimated population
Guinea	Boffa	2012–13	1166*	Gpg	169	140,000
Côte d’Ivoire	Bonon	2014	283	Gpp	19	80,000
Côte d’Ivoire	Sinfra	2014	341	Gpp	5	65,000
Côte d’Ivoire	Boblénou	2016	192	Gpg	0	1000
Burkina Faso	Gaoua	2014	729	Gpg, Gt	0	40,000

α Data provided by the WHO atlas for HAT [37].

* Previously described in [10].

Gpg = *Glossina palpalis gambiensis*; Gpp = *Glossina palpalis palpalis*; Gt = *Glossina tachinoides*.

### Trypanolysis test

All samples were sent to the Centre International de Recherche-Développement sur l’Élevage en zone Subhumide (CIRDES, Bobo-Dioulasso, Burkina Faso) to be tested by TL, as previously described [25, 39]. Because of a limited volume of plasma, TL was only performed using a cloned population of *T.b. gambiense* VAT LiTat 1.3. Briefly, 25 μL of plasma were mixed with 25 μL of guinea pig serum, to which 50 μL of a suspension with 10^7^ trypanosomes/mL prepared from infected mouse blood were added. After 90 min of incubation at ambient temperature, the suspension was examined by microscopy (×400). TL was considered positive when more than 50% of the trypanosomes were lysed.

### Statistical analysis

Statistical analysis were performed with R (Version 3.5.0 (2018-04-23) Ed. R Foundation for Statistical Computing, Vienna, Austria. http://www.R-project.org). The non-parametric Spearman’s rank test was used to assess the correlation between the percentages of positivity in the different serological tests and HAT endemicity (number of HAT cases/10,000 inhabitants/year calculated over the 2010–2014 period).

## Results

Results of serological (CATT and TL) and parasitological tests in all 2711 specimens are presented in Table 2 for each study site. No TL LiTat 1.3 positive results were observed among the samples from Gaoua (Burkina Faso) and Boblénou (Côte d’Ivoire). Fifty-two people, all from *gambiense*-HAT endemic foci in Guinea and Côte d’Ivoire, were positive for TL LiTat 1.3. The seven confirmed HAT cases, diagnosed in Guinea, were all positive in TL LiTat 1.3.

**Table 2:** Number of positive persons in microscopy, CATT and/or immune trypanolysis. Percentages are given between brackets.

Country	Area/Focus	*N*	T+	CATTB+	CATTP+	TL 1.3+	CATTB+/TL1 .3+	CATTP+/TL 1.3+	CATTB+/TL 1.3−	CATTB−/TL 1.3+
Guinea	Boffa	1166	7 (0.6)	34 (2.9)	10 (0.9)	44 (3.8)	15 (1.3)	10 (0.9)	19 (1.6)	29 (2.5)
Côte d’Ivoire	Bonon	283	0	12 (4.2)	6 (2.1)	6 (2.1)	1 (0.3)	1 (0.3)	11 (3.9)	5 (1.8)
Côte d’Ivoire	Sinfra	341	0	7 (2.1)	4 (1.2)	2 (0.6)	0	0	7 (2.1)	2 (0.6)
Côte d’Ivoire	Boblénou	192	0	1 (0.5)	0	0	0	0	1 (0.5)	0
Burkina Faso	Gaoua	729	0	6 (0.8)	4 (0.5)	0	0	0	6 (0.8)	0

*N* = number of persons sampled; CATT = Card Agglutination Test for Trypanosomiasis; CATTB = CATT on blood; CATTP = CATT on plasma; T+ = positive by parasitological examinations; TL = immune trypanolysis; 1.3 = variant antigen type (VAT) LiTat 1.3; + = positive test; − = negative test.

**Figure 1:**
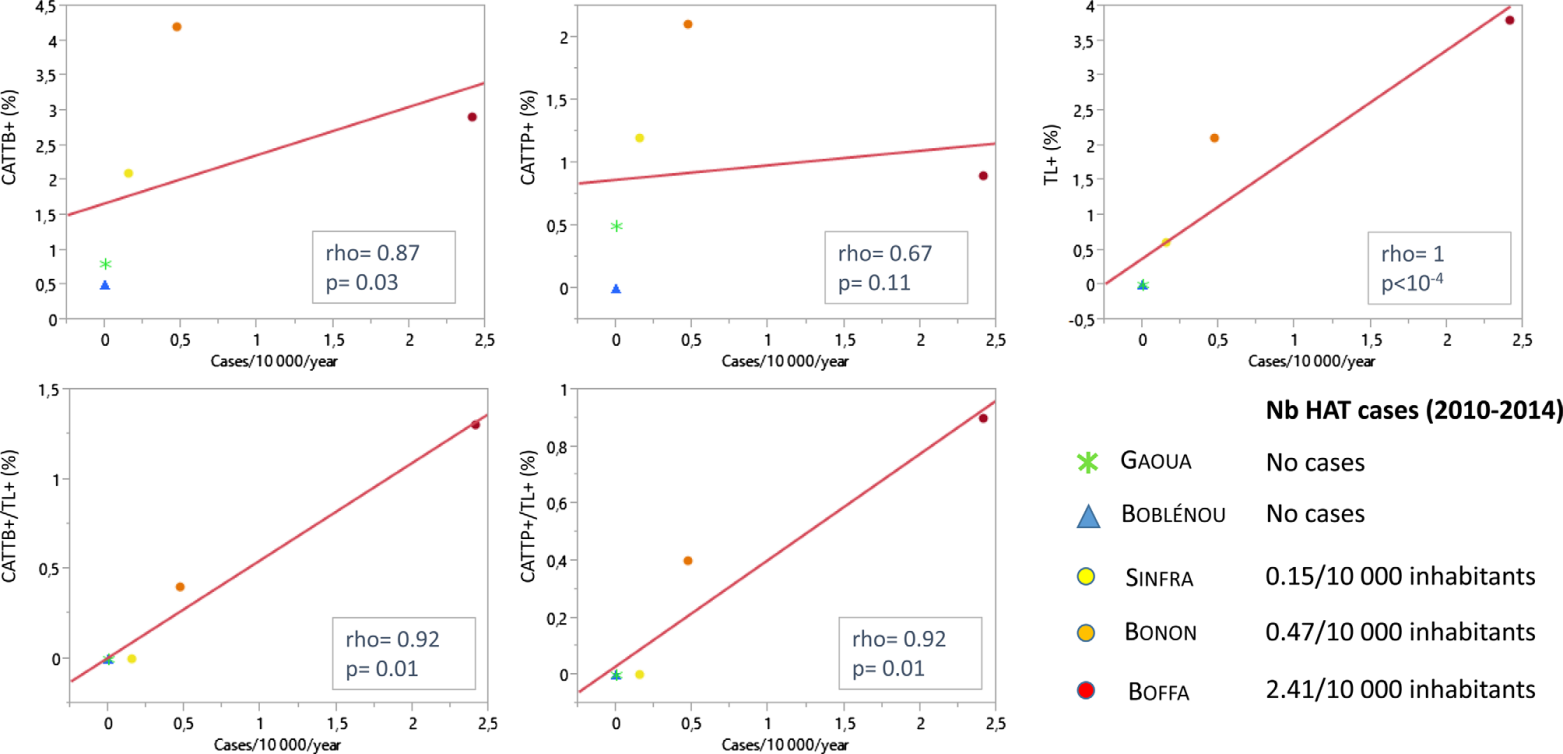
Relationship between the estimated annual *gambiense*-HAT incidence and the positivity of serological tests. The annual incidence is expressed as the mean number of reported HAT cases per 10,000 inhabitants calculated over the 2010–2014 period. CATT = Card Agglutination Test for Trypanosomiasis; CATTB = CATT on blood; CATTP = CATT on plasma; TL = Immune Trypanolysis LiTat 1.3; + = positive test; − = negative test. Correlation between variables were assesed by the Spearman’s rank test.

Figure 1 shows the relationship between the estimated HAT endemic levels and the percentage of positivity for serological tests and combinations thereof (CATTB, CATTP and TL). Only a moderate correlation was observed between HAT endemic levels and the percentage of positivity in CATTB (rho = 0.87; *p* = 0.03) in the population. The correlation was even lower and not significant when CATTP was considered (rho = 0.67; *p* = 0.1). Better correlations were observed between HAT endemic levels and the percentage of both CATTB+/TL+ and CATTP+/TL+ individuals (rho = 0.92; *p* = 0.01). However, these parameters did not discriminate Sinfra, where sporadic cases have been detected in the last 5 years, from Gaoua and Boblénou, where no cases were reported over the last 20 years. In contrast, the percentage of TL positivity alone correlated well with the epidemiological situation observed at each study site (rho = 1; *p* < 10^−4^), classifying the five sites correctly according to their HAT endemicity, increasing from 0% where no cases were reported between 2010 and 2014 to 3.8% in the Boffa focus in Guinea.

## Discussion

In this study, we explored the epidemiological significance of TL in different contexts regarding the current situation of *gambiense*-HAT in Burkina Faso, Côte d’Ivoire and Guinea. An original result of this paper is that for the first time, TL was performed at the population level whereas before it was mainly used to assess the status of serological suspected cases. In all endemic foci, the percentage of TL positivity was much higher than the estimated prevalence of parasitologically confirmed HAT patients and was significantly correlated with the HAT endemic level calculated over the 2010–2014 5-year period. In contrast, CATTB and CATTP that are routinely used for the serological testing of endemic populations, were poorly correlated with HAT endemicity. Importantly, this study shows that in all endemic foci, a number of individuals testing negative to the CATT are positive to the TL test (2.5%, 1.8% and 0.6% in Boffa, Bonon and Sinfra, respectively). These individuals could be either previously cured HAT patients or individuals who self-cured from the infection as the TL test is known to remain positive for years (up to 10 years in some HAT patients after cure), in contrast to the CATT that usually resolves within months in most patients after treatment [23, 27]. In contrast to the CATT that can be considered as an individual marker of active infection, positivity to the LiTat 1.3 trypanolysis test appears to capture part of the population history regarding *T.b. gambiense* transmission and population exposure. At the population level, TL is thus a marker of the intensity of transmission that occurred earlier and is therefore linked to the mean prevalence levels calculated over the last 5-year period.

This is not the case with CATT that becomes negative more quickly (within months after cure) and is known to have low positive predictive values especially in low endemic areas [9, 38]. During this study, none of the seven CATTB-positive individuals identified in Sinfra were positive to TL, whereas two TL-positive individuals were identified among CATTB-negative subjects. This suggests that, at least in the low endemic context of the present study, the percentage of TL positivity in the whole population is more informative than the percentage of TL positives among the CATTB positives. The surveillance strategies implemented in countries with no or very few reported HAT cases and with limited parasitology capacity, are currently based on serological testing of clinical suspected cases in a few sentinel sites. For all positive subjects, a blood sample is collected on filter paper and sent to a reference laboratory for TL testing. Only TL+ individuals are then examined parasitologically and a survey is organised in their close environment. In Benin and Togo for example, where such strategies are in place, only a few serological suspected cases are identified each year. The chance of finding an individual positive to TL is thus unlikely as the percentage of positivity in serological suspects is very low in foci with low endemic profiles [25]. Collecting randomly a few hundred blood samples on filter paper from endemic areas could be complementary to the existing passive surveillance system to better characterise the HAT endemicity status in these areas. For this study, TL testing was made on frozen plasma samples. Because TL performed on filter paper was shown to be less sensitive [6], analysing such samples could lead to a slight underestimation of the prevalence of TL positivity in the population. Nevertheless, collecting frozen plasma samples on a large scale may be challenging (both financially and logistically) in many context, whereas collecting blood on filter paper (a strategy currently in place for the passive surveillance of HAT in low endemic foci), is much easier and less costly, and may thus make it possible to reach better population coverage.

Although the analysis of the relationship between the percentage of TL positivity in the population and HAT prevalence needs to be supplemented by adding more foci to refine the correlation plots, we speculate from the data presented here that a percentage of TL positivity below 2% corresponds to areas where the elimination as a public health problem has been reached. On the one hand, the duration of TL positivity after successful cure [23, 27] may be regarded as a limitation, as the percentage of TL positivity may take time to decline (e.g., in Bonon), even though transmission has been severely impacted, but on the other hand, absence of TL positivity in the population (e.g., in Boblénou or Gaoua) appears to be a strong indication that transmission was indeed interrupted some years ago. In addition to being a sensitive and specific test for individual HAT diagnosis, TL thus also appears to be a promising biomarker to monitor the elimination of *gambiense*-HAT at the population level, especially in areas where the interruption of transmission needs to be validated.

This study was based on the analysis of five unique datasets for which TL results were available for the whole population screened with CATT during active case detection. Because these surveys were not initially designed to test TL as a population marker of transmission, this study has some limitations. One is that the sampling is limited and restricted to West Africa. In Central Africa, where some *T.b. gambiense* strains are suspected to lack LiTat 1.3 expression [17], inclusion of other variants in the TL may be needed before considering the implementation of TL as a tool to monitor HAT elimination at the continental scale. Another limitation is that we were not able, in the present study, to take into account covariates such as age and gender that could be confounding factors in the analysis. Even though we do not expect large differences between the different study sites in terms of age or gender distribution, the present exploratory analysis should be validated by a larger and specifically designed sampling strategy implemented in a larger number of foci.
